# GFI1 regulates chromatin state essential in human endothelial‐to‐haematopoietic transition

**DOI:** 10.1111/cpr.13244

**Published:** 2022-05-03

**Authors:** Baoqiang Kang, Tian Zhang, Ke Huang, Tianyu Wang, Yuhang Li, Yuchan Mai, Jinbing Li, Shiying Dang, Zhishuai Zhang, Wenhao Huang, Junwei Wang, Minghui Gao, Yi Wang, Guangjin Pan

**Affiliations:** ^1^ Department of Regenerative Medicine, School of Pharmaceutical Sciences Jilin University Changchun China; ^2^ CAS Key Laboratory of Regenerative Biology, Guangzhou Institutes of Biomedicine and Health Chinese Academy of Sciences Guangzhou China; ^3^ Guangdong Provincial Key Laboratory of Stem Cell and Regenerative Medicine South China Institute for Stem Cell Biology and Regenerative Medicine, Guangzhou Institutes of Biomedicine and Health, Chinese Academy of Sciences Guangzhou China; ^4^ University of Chinese Academy of Sciences Beijing China; ^5^ Medical Research Center, People's Hospital of Longhua Shenzhen China; ^6^ Institute for Stem Cell and Regeneration Chinese Academy of Sciences Beijing China; ^7^ The Seventh Affiliated Hospital, Sun Yat‐sen University Shenzhen China

## Abstract

**Objectives:**

During embryonic haematopoiesis, haematopoietic stem/progenitor cells (HSPCs) develop from hemogenic endothelial cells (HECs) though endothelial to haematopoietic transition (EHT). However, little is known about how EHT is regulated in human. Here, we report that GFI1 plays an essential role in enabling normal EHT during haematopoietic differentiation of human embryonic stem cells (hESCs).

**Results:**

GFI1 deletion in hESCs leads to a complete EHT defect due to a closed chromatin state of hematopoietic genes in HECs. Mechanically, directly regulates important signaling pathways essential for the EHT such as PI3K signaling.etc.

**Conclutions:**

Together, our findings reveal an essential role of GFI1 mediated epigenetic mechanism underlying human EHT during hematopoiesis.

## Graphical abstract

We found that GFI1 is essential for the generation of human haematopoietic progenitor cells (HPCs) through endothelial‐to‐haematopoietic transition (EHT). *GFI1* deletion in human embryonic stem cells (hESCs) resulted in the absence of HPC commitment. Mechanically, GFI1 promoted the EHT process through directly binding and activating the PI3K signalling. In contrast, in mouse haematopoiesis, *Gfi1* deletion alone did not significantly impair EHT. Our findings not only uncovered the essential role and mechanism of *GFI1* in regulating human haematopoiesis but also highlighted the significance of hPSCs in modelling human physiology.

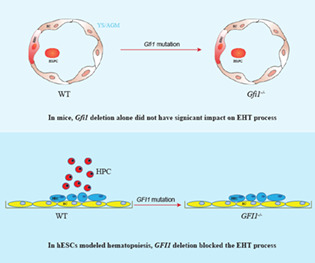

## 1 INTRODUCTION

During development, haematopoietic stem cells (HSCs) with multi‐lineage and long‐term repopulating potential are generated in the aorta‐gonad‐mesonephros (AGM) region.^1^, ^2^ Studies in mice and zebrafish demonstrated that haematopoietic stem or progenitor cells (HSPCs) are produced from hemogenic endothelial cells (HECs) through the endothelial‐to‐haematopoietic transition (EHT) process,^3^, ^4^, ^5^ during which the flattened endothelial cells gradually acquire the morphology and characteristics of haematopoietic cells.

Understanding the underlying mechanisms to control EHT could benefit the in vitro generation of HSPC from human pluripotent stem cells (hPSCs), including human induced pluripotent stem cells (hiPSCs) and human embryonic stem cells (hESCs). To date, a panel of signalling pathways and factors was found to be involved and regulate EHT. For instance, the signalling pathways such as Notch,^6^ retinoic acid^7^ and TGF‐beta^8^ were shown to be crucial in HSC development from HECs. In addition, transcription factors such as *Gata2* also play a central role to ensure EHT as *Gata2* deletion results in EHT blockage in mice.^9^, ^10^, ^11^ Other critical factors like *Ncor2* ^12^ and *Mellt3* ^13^ also play important roles to regulate EHT. Deletion of growth factor independent‐1(Gfi1) and Gfi1b led to the impairment of EHT in mice.^14^ However, deletion of Gfi1 or Gfi1b alone did not block the HSPC generation, indicating that these two proteins functionally compensate with each other during haematopoiesis.^14^, ^15^ At the mean time, how human EHT is regulated remains poorly understood due to limited research material. We previously reported a chemically defined, mono‐layer approach to differentiate hPSCs into HSPCs, which recapitulates major developmental stages during embryogenesis, such as mesoderm, HEC and EHT.^16^ This defined haematopoietic differentiation protocol provides an in vitro model to understand human EHT. Indeed, we revealed an essential role of vitamin C in regulating EHT based on this model, and this finding was further confirmed in the in vivo zebrafish model.^16^ We also identified CD44 as a marker for nascent multipotent HSPCs from hPSCs and this finding again was confirmed in single‐cell data generated directly from human embryos.^17^

Here, in this report, we investigate the role of *GFI1* in human EHT based on our defined protocol for hPSC haematopoietic differentiation. We found that in contrast to its role identified in mouse model, *GFI1* deletion alone in hESCs sufficiently leads a complete EHT defect during haematopoietic differentiation, independent of *GFI1B*. Mechanistically, GFI1 maintains the open chromatin state of genes important for EHT, such as PI3K signalling in HECs to enable EHT. Our findings reveal an essential role of GFI1‐mediated epigenetic mechanism underlying human EHT during haematopoiesis.

## 2 MATERIALS AND METHODS

### 2.1 Cell lines

The hESC lines used in this study include H1 hESCs, H1‐*GFI1* ^*−/−*^, H1‐*GATA2* ^*w/eGFP*^, H1‐*GATA2* ^*w/eGFP*^‐*GFI1* ^*−/−*^, H1‐*GFI1* ^*w2×/flag*^ and H1‐GATA2^w/eGFP^‐GFI1^−/−^‐GFI1(FUW). H1 hESCs were obtained from WiCell Research Institute, and other cells lines above were derived in our lab through gene editing from the H1 hESCs.

### 2.2 Cell culture

The hESCs, including H1 and their derivatives, were maintained in mTeSR1 medium (Stemcell Technologies) on matrigel‐coated plates (Corning), at 37°C and with 5% CO_2_. The hESCs were passaged every 3–4 days with 0.5 mM ethylene Diamine Tetra acetic Acid.

### 2.3 Gene targeting

Gene knockout of *GFI1* was performed by the CRISPR/Cas9 system in H1 and H1‐*GATA2* ^*w/eGFP*^ cell lines. The pX330‐U6‐PGK‐hSpCas9 vector, containing the sgRNA targeting the Exon 2 or Exon 6 of GFI1 gene designed by sgRNA Designer (https://portals.broadinstitute.org/gpp/public/analysis-tools/sgrna-design), was electroporated into hESCs, using Nucleofector™ 2b Device (Lonza). After that, the cells were seeded onto the matrigel‐coated 6‐well plate in the presence of ROCK inhibitor—Y27632 (5 μM, Sigma). After clonal expansion for about 10 days, each individual hESC clone was picked for genotype analysis. Then, we used PCR and Sanger sequencing to detect the gene mutation of each clone. In detail, the PCR products of the gene targeting region were ligated to the pCE2‐TA‐Blunt‐Zero cloning vector (Vazyme). Then, the ligation products were transformed into DH5α Competent Cells. After that, individual *E.coli* clones were picked for Sanger sequencing to analyse the genotype of each hESCs clone. Finally, we harvested 3 clones with GFI1 mutation in double alleles. These *GFI1*‐mutated clones were further confirmed by the RT‐qPCR. Then, we performed fluorescence‐activated cell sorting (FACS) analysis, immunostaining, teratoma formation, and karyotype analysis to analyse the pluripotency of these *GFI1*‐mutated clones.

Also, the 2 × flag‐tag knockin into the GFI1 stop code upstream region was performed using the same strategy as the knockout above. Briefly, in addition to the px330, we used a targeting vector, which contains two homology arms about 1 kb upstream and downstream of the end of the GFI1 last exon, respectively. In addition, there was a 2 × flag tag and a loxP‐flanked PGK puromycin cassette between the homology arms. After electroporating px330 and targeting vector, the cells were selected with puromycin (0.5 μg/ml), and the positive individual clones were picked for further screening. Then, the positive clones were verified and further electroporated with 400 ng Cre‐mRNA to remove the loxp‐flanked PGK‐puromycin cassette. Thereafter, the H1‐*GFI1* ^*w2×/flag*^ clone was expanded for the pluripotency analysis as discussed above.^18^

### 2.4 Teratoma formation

The teratoma‐formation experiments have been reviewed and approved by IACUC at Guangzhou Institutes of Biomedicine and Health, Chinese Academy of Sciences. In detail, approximately 1 million hESCs suspended in 50% cold matrigel (BD, diluted by DMEM/F12 medium [Hyclone]) were subcutaneously injected into NOD‐SCID mice (Vital River). After 6‐to‐8 weeks, the teratoma was harvested and fixed in 4% paraformaldehyde (PFA). Furthermore, the haematoxylin/eosin (H&E) staining was performed to detect the three germ‐layer formation potential of the hESCs.

### 2.5 Haematopoietic differentiation

Before haematopoietic differentiation, hPSCs were dissociated by Accutase (Sigma) and plated on growth factor‐reduced Matrigel (Corning)‐coated plates with thiazovivin (0.1 μM, Selleck). Firstly, at day 0, 40 ng/ml of BMP4 (Peprotech), 30 ng/ml of ACTIVINA (Sino Biological Inc.), 20 ng/ml of bFGF (Sino Biological Inc.), 6 μM CHIR99021 (Selleck) and 10 μM LY294002 (Selleck) were added to the basic medium (BM, mimics of the CustommTeSR1) of Dulbecco's‐modified Eagle's medium/F‐12 (GIBCO) supplemented with 1% insulin–transferrin–selenium (GIBCO), 70 μg/ml of vitamin C (Sigma). Second, 30 ng/ml of BMP, 1 μM A8301 (Selleck) and 2 μM IWR‐1‐endo (Selleck) were added to the BM on day 1. Then, on days 2–4 of differentiation, 40 ng/ml of vascular endothelial growth factor (Sino Biological Inc.) and 50 ng/ml of bFGF were added to the BM. Finally, 40 ng/ml of vascular endothelial growth factor, 50 ng/ml of bFGF, 10 μM SB431542 (Selleck), 10 ng/ml of stem cell factor (Peprotech), 50 ng/ml of thrombopoietin (Sino Biological Inc.), 10 ng/ml ofinterleukin 3 (Sino Biological Inc.) and 50 ng/ml of interleukin 6 (Sino Biological Inc.) were added in the BM at days 4–6 of differentiation and further haematopoietic commitment and maturation.

### 2.6 CFU assay

Single cells of the certain cell number were suspended in the 100 μl of Iscove's modified Dulbecco's medium (Gibico) supplemented with 2% FBS (Biological Industries) and then mixed with 1 ml of Methocult H4435 (Stem Cell Technologies) in 35‐mm ultra‐low attachment plates (Stem Cell Technologies). After the cell culture at 37°C and with 5% CO_2_ of 14 days, the CFUs were classified and counted according to their morphology.

### 2.7 Virus production and transduction

*GFI1* open‐reading frame was cloned into TetO‐FUW‐EBHX‐P2a‐EGFP‐T2a‐Puro lentiviral vectors. Lentiviral particles were produced by transfecting 293 T cells with the packaging plasmids pMDL, VSVG and pRSV‐Rev. Viruses were harvested at 36 and 60 h after transfection and concentrated by ultracentrifugation at 50,000 *g* for 2.5 h at 4°C. Viruses were resuspended with DMEM/F12 medium (Gibico). Transduction was carried out in 6‐well plates with FUW‐rtTA when the confluent of the cell line H1‐GFI1^−/−^ was about 40%. Doxycycline (dox) (Sigma‐Aldrich) was added at a concentration of 20 μg/ml for transgene induction. After the 48 h of Dox adding, puromycin (0.5 μg/ml) was applied to select positive individual clones.

### 2.8 RT‐qPCR

Total RNA was isolated by the TRIzol (Sigma) following the manufacturer's instructions. Then, the RNA was reverse transcribed into cDNA by the HiScript® III RT SuperMix for RT‐qPCR (Vazyme). The RT‐qPCR was performed on a CFX96 machine (Bio‐Rad) with the SYBR Green Mix (Vazyme). PCR cycle conditions were at 95°C for 10 min for denaturation, 40 cycles of 95°C for 10 s, 60°C for 10 s and 72°C for 20s. All experiments were done in triplicate. Primers used in this study were listed in Supporting Information Table S1.

### 2.9 Fluorescence‐activated cell sorting

The cells were digested into single cells by accutase (Sigma) and suspended in PBS with 2% FBS. Then, cell suspensions were incubated with the antibody for 30 min at 4°C. After the incubation, the marker expressions in this study were analysed by the CytoFLEX Flow Cytometer (Beckman). For cell sorting, cells were sorted by the Moflo (Beckman). Antibodies used in this study were listed in Supporting Information Table S2.

### 2.10 Immunostaining

The cells were incubated with 4% paraformaldehyde at room temperature for 2 min. Then, cells were washed with PBS and incubated with the primary antibody in PBS with 10% bovine serum albumin (BSA) and 0.3% TritonX‐100 overnight at 4°C. Then, the cells were washed twice and further staining with secondary antibody for 1 h in PBS with 1% BSA at room temperature. After the incubation, nuclei were counterstained with 4′,6‐diamidino‐2‐phenylindole (DAPI; Sigma) for an additional 5 min. Finally, the cells were imaged with a Confocal laser scanning microscope (Zeiss LSM 710).

### 2.11 RNA‐Seq

H1‐*GATA2* ^*w/eGFP*^ and H1‐*GATA2* ^*w/eGFP*^‐*GFI1* ^*−/−*^ HECs at day 4 of differentiation were lysed with TRIzol (Sigma) to isolated total RNA, and sequencing libraries were prepared by the TruSeq RNA Sample Prep Kit (Illumina) under the manufacturer's recommendations. The samples were run on a NextSeq system with NextSeq 500 Mid Output kit (Illumina). RNA‐Seq data were processed as follows. Reads were aligned to an index generated from the Ensembl transcriptome version 74 (hg38), using HISAT2, and gene expression was analysed with SAMtools and htseq‐count, normalised with EDASeq. A threshold of at least 20 average row read counts was used to filter lowly expressed transcripts. Differential expression was performed using DESeq2 and genes were considered significant if they had a BenjaminiHochberg corrected *p* value <.05. Gene ontology was performed using clusterProfiler; heatmap was prepared using pheatmap and Gene set enrichment analysis was analysed using the GSEA software.

### 2.12 ATAC‐Seq

ATAC‐seq and data analysis were performed as follows. 50,000 cells of each sample were collected to generate DNA libraries with a Nextera DNA library preparation kit (Illumina) according to the manufacturer's recommendations. Then, the DNA libraries were run on a NextSeq system with NextSeq 500. Then, the sequencing data were mapped on to human genome (UCSC hg38) using bowtie2. Signals were compiled using MACS2 call peak, and MACS2 was used to call open peaks. Signal density heatmap and profile were plotted using deep Tools, motifs were found using homer and gene ontology analysis was performed using DAVID.

### 2.13 CUT&Tag

CUT&Tag DNA library was prepared using Hyperactive pG‐Tn5 Transposase kit (Vazyme), following the manufacturer's instructions, and the DNA libraries for sequencing by NextSeq 500. All sequencing data were mapped onto hg38 using bowtie2; peaks were called using MACS2; and gene ontology analysis was performed using DAVID.

## 3 RESULT

### 3.1 GFI1 is essential in HPC generation during hESC differentiation

**FIGURE 1:**
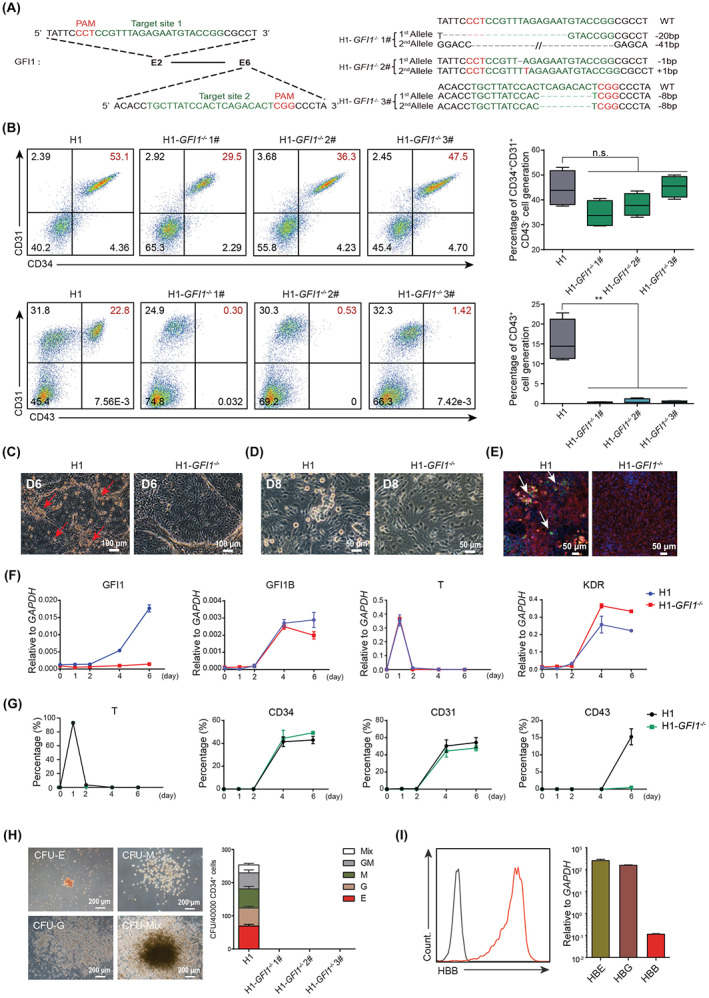
GFI1 deletion blocked the HPC generation from hESCs. (A) Schematic representation of the sgRNA and corresponding gene knockout strategy in Exon2 and Exon6 of the *GFI1* locus in H1 hESCs (*left*). The genotype of the mutated clones was listed in the right panel. (B) FACS analysis of the CD34^+^CD31^+^ ECs and CD43^+^ HPCs derived from H1‐*GFI1* ^*−/−*^ and WT H1 at day 6 of the haematopoietic differentiation. Statistics was determined using unpaired two‐tailed Student's *t*‐tests, **p < .01. The data represent mean ± SD from three independent biologic replicates (*n* = 3). (C, D) Morphology of the EHT at day 6 of the haematopoietic differentiation. Red arrow, emerged haematopoietic cells/clusters. Scale bar: 100 or 50 μm. (E) Immunofluorescence staining analysis of HPCs derived from hESCs at day 6 of haematopoietic differentiation. Blue, DAPI; Green, CD43^+^ hHPCs; Red, CD31^+^ hECs; White arrow, CD43^+^ hHPCs. Scale bar: 50 μm. (F) RT‐qPCR analysis of the indicated gene expression during the haematopoietic differentiation of the H1 and H1‐*GFI1* ^*−/−*^ 1# cells. These data represent mean ± SD from three independent replicates (*n* = 3). (G) FACS analysis of the percentage of T^+^, CD31^+^, CD34^+^ and CD43^+^ cells during the haematopoietic differentiation of the H1 and H1‐*GFI1* ^*−/−*^ 1# cells. These data represent mean ± SD from three independent technical replicates (*n* = 3). (H) Representative pictures of the indicated CFUs (*left*) and statistics of the CFUs formed from the 40,000 CD34^+^ cells derived from the indicated cells (*right*). Scale bar: 200 μm. E, erythroid; G, granulocytes; M, monocytes; GM, granulocyte and monocyte; Mix, mixed erythro‐myeloid. (I) FACS analysis of HBB expression in erythroid from the CFUs (*left*); RT‐qPCR analysis of HBB, HBE and HBG genes in erythroid from the CFUs (*right*)

To investigate the role of *GFI1* in human haematopoiesis, we generated *GFI1* deletion in H1 hESCs using the CRISPR/Cas9 gene editing. Small‐guide RNAs (sgRNAs) were designed to target Exon 2 and Exon 6, respectively (Figure 1A). After clone selection, we generated three mutated hESC clones in both alleles of *GFI1* (H1‐*GFI1* ^*−/−*^) (Figure 1A). These *GFI1* ^*−/−*^‐mutated clones displayed normal hPSC phenotype in terms of pluripotent marker expression and teratoma‐formation potential (Figure S1) compared to the wild‐type (WT) H1 hESCs. However, three *GFI1* ^*−/−*^‐mutated clones showed alomost complete defect in generation of CD43^+^ HPCs during haematopoietic differentiation of hESCs based on our previously reported monolayer differentiation protocol^16^ (Figure 1B). While the CD34^+^CD31^+^CD43^−^ endothelial cells were relatively normal in *GFI1* mutant clones (Figure 1B), indicating *GFI1* deletion leads an EHT defect during human hemotopoietic differentiation. Indeed, both morphology and immunostaining confirmed the absence of EHT in *GFI1*‐delected cells (Figure 1C–E **).** Further RT‐qPCR analysis comfirmed the loss of *GFI1*, but not *GFI1B* expression during the haematopoietic differentiation of *GFI1*‐deleted cells, and showed that expression of the early mesoderm gene *T* and hematovascular mesoderm gene *KDR* were not affected in *GFI1*‐deleted cells (Figure 1F). FACS analysis further comfired *GFI1*‐deleted cells underwent normol mesoderm, endothelial commitment but HPC defect based on markers for mesoderm (T), endothelial (CD31) or HPC (CD43) (Figure 1G).^19^ Indeed, loss of CD43^+^ HPCs led to the absence of colony‐forming unit (CFU) of the H1‐*GFI1* ^*−/−*^ cells (Figure 1H). In particular, the CFU‐E could express both the fetal and adult globins (Figure 1I), hinting that hESCs with *GFI1* mutation might lose the primitive and definitive haematopoiesis potential.^20^ Together, these data indicate *GFI1* deletion alone leads to haematopoietic defect in human differentiation.

### 3.2 GFI1 is required for EHT process

**FIGURE 2:**
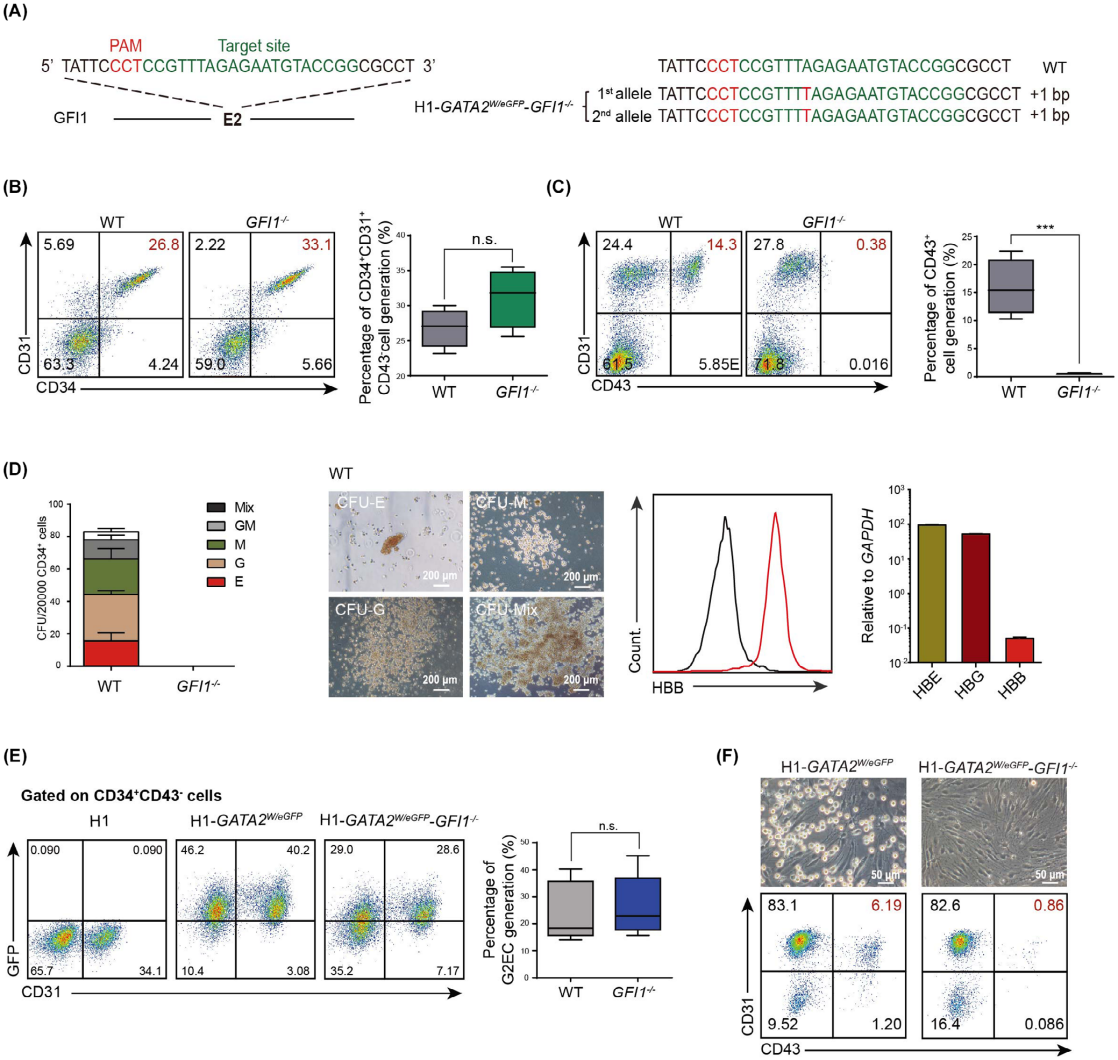
GFI1 deletion blocked the EHT process. (A) Schematic representation of the sgRNA and corresponding gene knockout strategy in Exon2 of the *GFI1* locus in H1‐*GATA2* ^*w/eGFP*^ hESCs (*left*); the genotype of the mutated clone was listed in the right panel. (B and C) FACS analysis of the CD34^+^CD31^+^ ECs and CD43^+^ HPCs derived from WT H1‐*GATA2* ^*w/eGFP*^ and H1‐*GATA2* ^*w/eGFP*^‐*GFI1* ^*−/−*^ cells at day 6 of the haematopoietic differentiation. Statistics was determined using unpaired two‐tailed Student's *t*‐tests; n.s., no significance; ***p < .001. These data represent mean ± SD from three independent biological replicates (*n* = 3). (D) From *left* to *right*: representative pictures of the indicated CFUs; statistics of the CFUs formed from the 20,000 CD34^+^ cells derived from the indicated cells; FACS analysis of HBB expression in erythroid from the CFUs; RT‐qPCR analysis of HBB, HBE and HBG genes in erythroid from the CFUs;. Scale bar: 200 μm. E, erythroid; G, granulocytes; M, monocytes; GM, granulocyte and monocyte; Mix, mixed erythro‐myeloid. (E) FACS analysis of the CD34^+^CD31^+^CD43^−^GATA2/eGFP^+^ HECs (G2ECs) derived from the H1‐*GATA2* ^*w/eGFP*^ and H1‐*GATA2* ^*w/eGFP*^‐*GFI1* ^*−/−*^ cells at day 4 of the haematopoietic differentiation. Statistics was determined using unpaired two‐tailed Student's *t*‐tests. The data represent mean ± SD from four independent biological replicates (*n* = 4). (F) Morphology (top) and FACS analysis (bottom) of the CD43^+^ HPC generation after the 4 days' culture of the sorted G2ECs from the indicated cells

To analyse the mechanisms underlying haematopoietic defect in *GFI1*‐mutated hESCs, we deleted *GFI1* in another hESC line with GATA2/eGFP knockin (H1‐*GATA2* ^*w/eGFP*^‐*GFI1* ^*−/−*^) (Figure 2A), as we have previously shown that GATA2/eGFP specifically labels HECs in hESCs differentiation. H1‐*GATA2* ^*w/eGFP*^‐*GFI1* ^*−/−*^showed normal hPSC characteristics in terms of pluripotent gene expression (Figure S2A–C), karyotype (Figure S2D) as well as teratoma formation (Figure S2E). Upon haematopoietic differentiaton, H1‐*GATA2* ^*w/eGFP*^‐*GFI1* ^*−/−*^ generated normal CD34^+^CD31^+^CD43^−^ endothelial cells, but impared CD43^+^ HPCs as well as no CFUs (Figure 2B–D). The percentage of GATA2/eGFP^+^ cells in CD34^+^CD31^+^CD43^−^ endothelial population(G2EC) that were enriched with HECs showed no substantial difference between H1‐*GATA2* ^*w/eGFP*^‐*GFI1* ^*−/−*^ and wild‐type cells (Figure 2E). However, the G2ECs derived from H1‐*GATA2* ^*w/eGFP*^‐*GFI1* ^*−/−*^ failed to undergo EHT upon replating into the EHT condition (Figure 2F), suggesting GFI1 is essential to ensure EHT rather than HECs commiting. In addition, we proved that re‐rexpression of *GFI1* in *GFI1* ^*−/−*^ cells substantially rescue the HPC generations, confirming that the impairment of EHT of *GFI1* ^*−/−*^ cells was indeed GFI1 dependent. (Figure S3B).

### 3.3 GFI1 sustained the haematopoietic program in HECs

**FIGURE 3:**
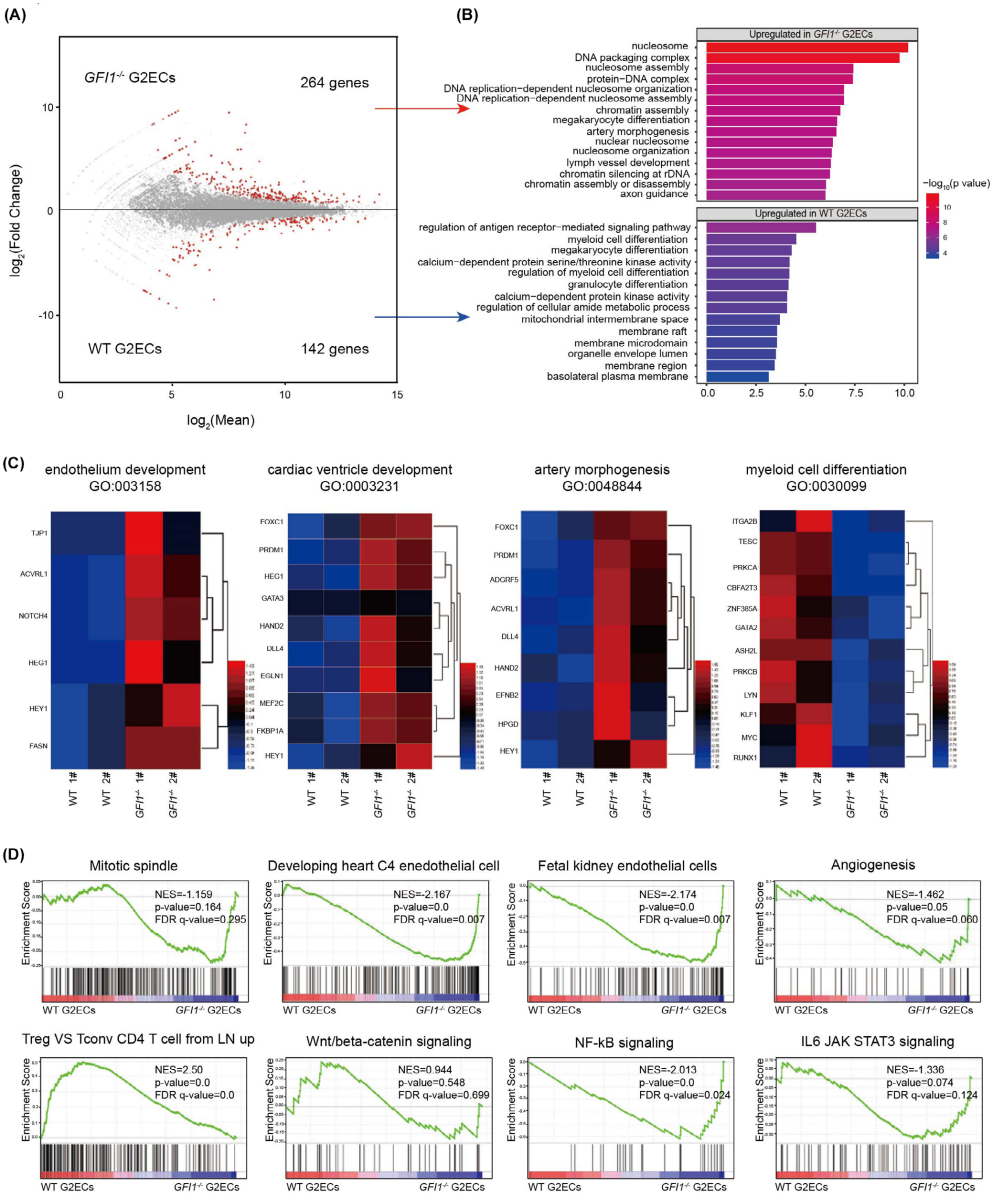
GFI1 sustained the haematopoietic program in HECs. (A) MA plot for genes expression in WT versus GFI1^−/−^ G2ECs. The *x*‐axis shows log2 mean; *y*‐axis shows log2 transformation of fold change and red points indicate significant genes. (B) Top Gene Ontology (GO) terms enriched in genes upregulated in GFI1^−/−^ G2ECs (*up*), and upregulated in WT G2ECs (*bottom*). (C) Heatmap of the gene expression of the indicated GO terms normalised by *Z* score. (D) GSEA analysis for WT and *GFI1* ^*−/−*^ G2ECs

To investigate the molecular mechanism underlying the functional deficiency in *GFI1*‐deleted HECs, we compared the transcriptional profile between the WT and *GFI1* ^*−/−*^ G2ECs through RNA‐seq (Figure 3A). 264 genes were up‐regulated *GFI1* ^*−/−*^ G2ECs and they were enriched the vascular biology and DNA replication‐related functions (Figure 3B). Meanwhile, 142 genes were down‐regulated in the *GFI1* ^*−/−*^ G2ECs and these genes are related to cell development (Figure 3B). For example, genes related to vascular and heart development, such as endothelium development (*TJP1*, *NOTCH4*, *HEG1*), cardiac ventricle development (*FOXC1*, *DLL4*, *EGLN1*) and artery morphogenesis (*ADGRF5*, *ACVRL1*, *EFNB2*) were up‐regulated in *GFI1* ^*−/−*^ G2ECs. The well‐known master regulators in haematopoiesis like *GATA2*, *KLF1*, *RUNX1*, etc. were down‐regulated in *GFI1* ^*−/−*^ G2ECs (Figure 3C). Further Gene Set Enrichment Analysis (GSEA) analysis also demonstrated the gain of accelerated cell cycle, endothelial/vascular and accelerated cell cycle program and loss of haematopoietic profile in *GFI1* ^*−/−*^ G2ECs (Figure 3D). In addition, we also identify the enrichment of NF‐kappaB and IL6/JAK/STAT3 signalling in *GFI1* ^*−/−*^ G2ECs (Figure 3D), suggesting that they might have a negative regulation in EHT. These data indicate *GFI1* deletion in HECs resulting elevated endothelial gene program and down‐regulated haematopoiesis program.

### 3.4 GFI1 promotes the chromatin accessibility on haematopoietic genes in HECs

**FIGURE 4:**
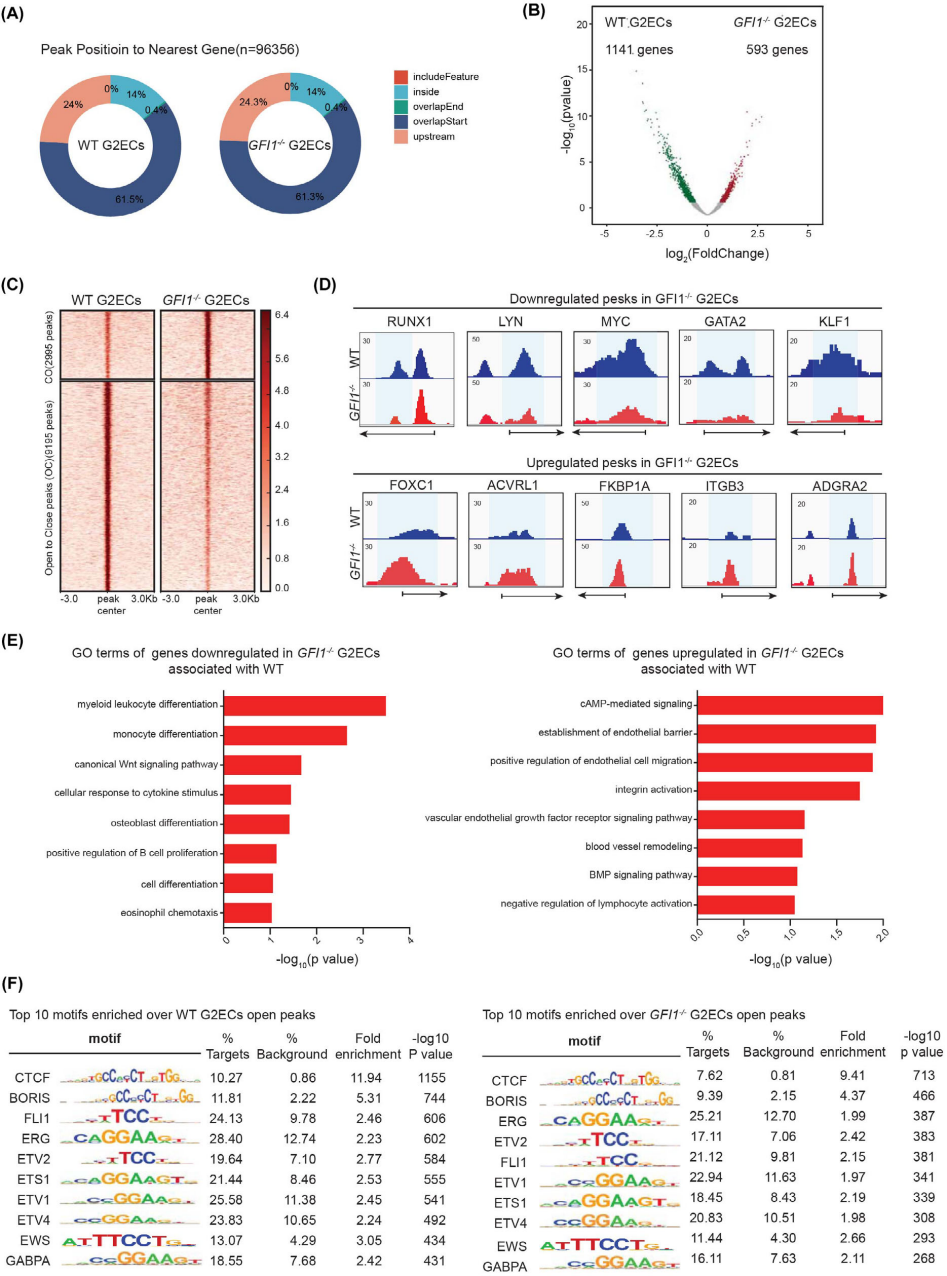
GFI1 promoted the chromatin accessibility on haematopoietic genes in HECs. (A) Distribution of the ATAC‐seq peaks in the genomic regions of the WT and *GFI1* ^*−/−*^ G2ECs. (B) Volcano plot of the accessible peaks of the WT and *GFI1* ^*−/−*^ G2ECs; *x*‐axis shows log_2_(fold change of the peak area); *y*‐axis shows log_10_(*p* value of peak); the blue dots represent differentially accessible peaks in WT G2ECs; the red points indicate differentially accessible peaks in *GFI1* ^*−/−*^ G2ECs. (C) ATAC density heat map of the differential opened and closed peaks in WT and *GFI1* ^*−/−*^ G2ECs. (D) Selected genomic views of the ATAC‐seq data at the indicated gene locus. (E) Selected GO terms enriched in genes down regulated (*left*) and up regulated (*right*) in *GFI1* ^*−/−*^ G2ECs compared to the WT G2ECs. (F) known motif enrichment analysis of the accessible regions in WT and *GFI1* ^*−/−*^ G2ECs cells by homer. Fold‐enrichment was calculated by target %/background %

To investigate how *GFI1* deletion leads expression change in HECs, we examined chromatin assessbility in WT and *GFI1* ^*−/−*^ G2ECs by transposase‐accessible chromatin with sequencing (ATAC‐seq) as chromatin accessibility change might precede the transcription.^16^ As expected, the detected peaks by ATAC‐seq were close to the gene bodies, especially the transcriptional start site (TSS, overlapStart) (Figure 4A). Substantial number of differential accessible chromatin regions were identified between the WT and *GFI1* ^*−/−*^ G2ECs (Figure 4B). These differential accessible peaks associated with 1141 genes down‐regulated and 593 genes up‐regulated in *GFI1* ^*−/−*^ G2ECs (Figure 4C). The down‐regulated haematopoietic genes like *RUNX1* and *FOXC1*, etc. showed obvious decreased chromatin accessibility in *GFI1* ^*−/−*^ G2ECs (Figure 4D). In general, blood cell development‐relgated gene showed decreased chromatin accessibility while endothelial genes showed increased chromatin accessibility upon *GFI1* deletion (Figure 4E). Lastly, the accessible chromatin regions promoted by *GFI1* were enriched in many haematopoietic regulatorary genes like *FLI1, ERG, ETV2, ETS1*, etc. (Figure 4F). Together, these data suggest that *GFI1* promotes chromatin accessibilities on haematopoietic genes in human HECs.

### 3.5 GFI1 regulates essential signalling pathways for EHT

**FIGURE 5:**
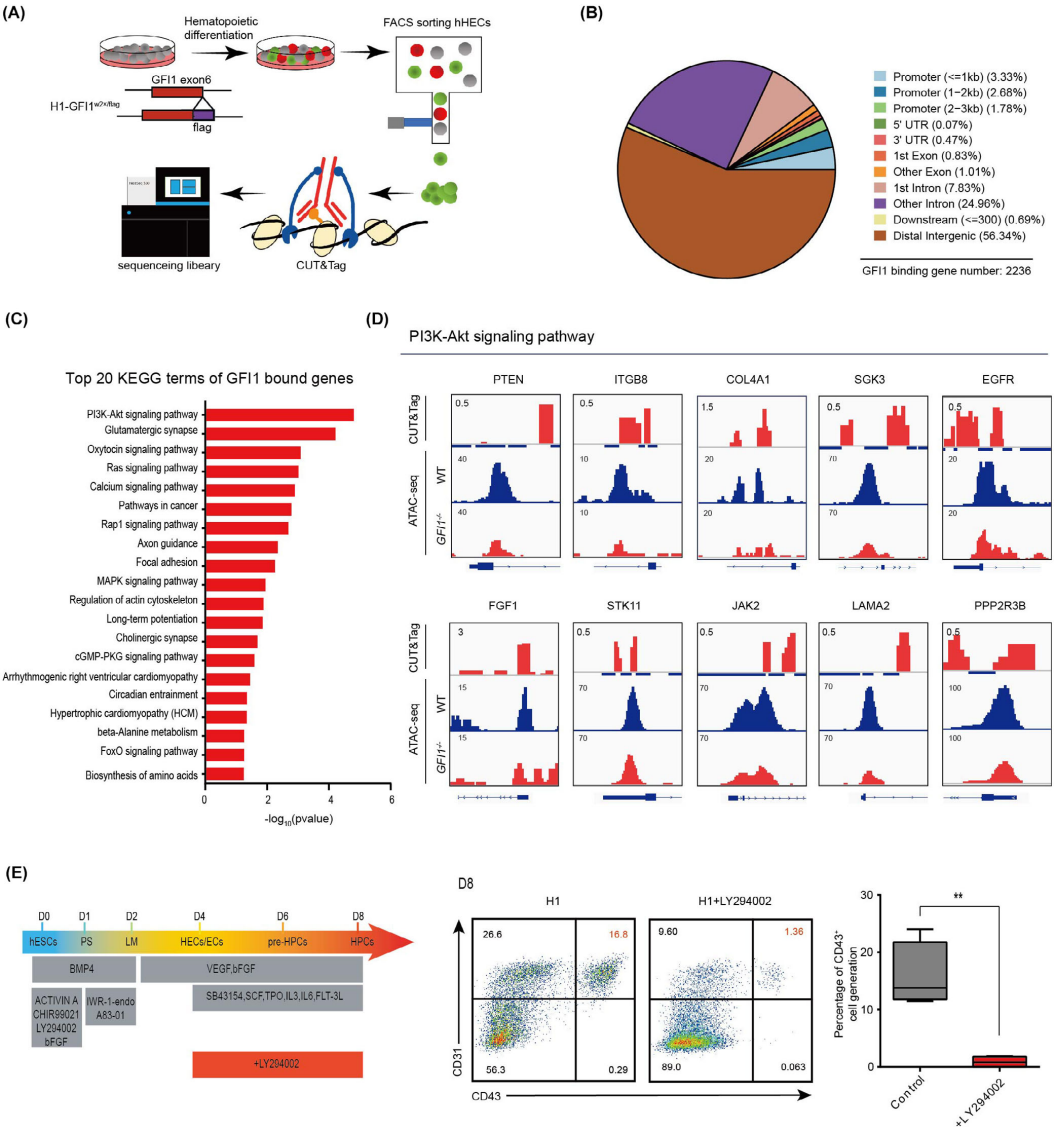
GFI1 regulated EHT process through activating PI3K signalling. (A) Work flow of the GFI1 gene binding analysis: Generating a GFI1‐flag‐knock in hES cell line using CRISPR‐Cas9 technology, and then sorting endothelial cells during hESCs blood differentiation by FACS, finally analysing its direct chromatin‐binding through CUT&tag (B) Distribution of the GFI1 binding peaks in the genomic regions detected by Cut&tag. (C) Top KEGG terms enriched of GFI1 binding genes. (D) Cut&tag and ATAC‐seq tracks of PI3K‐Akt signalling pathway associated genes. (E) Experimental design to analyse the influence of PI3K inhibition on HPC generation. The canonical PI3K inhibitor—LY294002 was added from day4 to day8 during haematopoietic differentiation. (F) FACS analysis of the CD43^+^ HPC generation from the indicated group at day 8 of the haematopoietic differentiation. Statistics was determined using unpaired two‐tailed Student's *t*‐tests, **p < .01. The data represent mean ± SD from three independent replicates (*n* = 3)

To examine the direct targets of GFI1 in regulating EHT, we analysed its direct chromatin‐binding targets in CD34^+^CD31^+^CD43^−^ endothelial cells sorted in hESCs blood differentiation through cleavage under targets and tagmentation (CUT&tag) analysis (Figure 5A). We identified total 2926 GFI1 binding peaks that assoaciated to 2236 anotated genes in the hESCs derived CD34^+^CD31^+^CD43^−^ endothelial cells (Figure 5B). These targets contain many signalling pathways involved in HSC maintenance, such as MAPK^21^ and FoxO^22^ signalling pathways (Figure 5C). Among them, the PI3K‐Akt signalling is the top 1 enriched pathway in GFI1 targets (Figure 5C). Moreover, the GFI1 bound PI3K‐Akt signalling‐related genes showed decreased chromatin accessibilities upon GFI1 deletion (Figure 5D). Notably, previous studies have shown that PI3K signalling was involved in HSC maintenance.^23^ Thus, we examined PI3K signalling in EHT process and showed that its inhibition blocked the HPC generation during haematopoietic differentiation of hESCs (Figure 5E). However, activating PI3K signalling by SC79 could not rescue EHT defect in GFI1^−/−^ cells, indicating GFI1 deletion might lead to defect in many other pathways essential for EHT (Figure S3A). Nonethless, these results indicated that GFI1 regulates essential signalling pathways for EHT process.

## 4 DISCUSSION

During early development, definitive HSPCs are generated from HECs through the well‐known EHT process that is demonstrated in various model systems like mice, zebrafish etc. However, how EHT is regulated in human remains largely unknown due to the limited research material. Differentiation of hPSCs to haematopoietic lineage cells in vitro might serve as a valuable model to understand human haematopoiesis. Based on hPSC differentiation model, couple pathways or regulators had been revealed to regulate human haematopoiesis, such as SOX17,^24^ HOX9^25^ and vitamin C.^16^ In our previous study, we established an approach to differentiate hPSCs into HSPCs in a chemically defined, mono‐layer condition that recapitulate major haematopoiesis developmental stages, such as mesoderm, HEC and EHT.^16^ In this report, based on this approach, we revealed that GFI1 plays an essential role in human haematopoiesis to enable normal EHT through maintaining chromatin accessibility in critical haematopoietic regulatorary genes such as PI3K signalling. Our findings highlight the important role of epigenetic machinism to ensure normal EHT, which has not been highly documented.

GFI1 has been reported to involve in epigenetic regulation through recruiting histone methyltransferases (G9),^26^ histone demethylase (LSD1)^15^ and histone deacetylases^27^ to repress target gene transcription. In mouse haematopoiesis, *Gfi1* deletion alone did not significantly impair EHT while deletion of both *Gfi1*and *GFib* lead severe EHT defect, as the Gfi1 proteins could recruit the chromatin‐modifying protein LSD1 to epigenetically silence the endothelial program in HECs.^15^ Consistently, a recent study reported that blocking GFI1 and/or GFI1b activity with a small molecule inhibitor of LSD1 could also abrogated all blood cell development from hiPSCs.^28^ While in this report, we further showed that GFI1 deletion alone led complete EHT blockage, indicating the differential requirement of GFI1 in different species. In fact, how exactly EHT accurs at the molecular level remains unclear. We failed to detect more genes with expression change in *GFI1* ^−/−^ HECs compared with WT HECs (Figure 3). Interstingly, *GFI1* ^−/−^ HECs exibit reduced chromatin assessbilies in those haematopoiesis regulatory genes such as *FLI1, ERG, ETV2 and ETS1* indicating the critical haematopoietic genes have been predisposed at the chromatin level in endothelial cells without significant transcriptional change before EHT. The critical factors such as GFI1 establish the predisposed haematopoietic gene program to ensure the subsequent EHT process for HSPCs generation.

## CONFLICT OF INTEREST

The authors declare no conflict of interest.

## AUTHOR CONTRIBUTIONS

Tian Zhang and Ke Huang designed the project. Baoqiang Kang, Tian Zhang and Ke Huang designed and performed the experiments, analysed the data and assisted the manuscript writing. Tianyu Wang analysed the RNA sequencing and ATAC sequencing data. Shiying Dang analysed the CUT&Tag data. Yuhang Li, Yuchan Mai and Jinbing Li assisted with the experiments. Zhishuai Zhang and Wenhao Huang performed the teratoma assay. Junwei Wang and Minghui Gao assisted the cell sorting. Yi Wang gave suggestions about experiments and the manuscript. Guangjin Pan conceived, supervised the whole study and wrote the manuscript.

## Supporting information


**FIGURE S1** (A) FACS analysis of the classical pluripotent marker expression (OCT4, SSEA4, TRA‐1‐60 and TRA‐1‐81) of the H1‐*GFI1* ^*−/−*^ 1#, 2# and 3# cells compared with H1. (B) Immunofluorescence staining analysis of the classical pluripotent marker expression (OCT4) of the H1‐*GFI1* ^*−/−*^ 1#, 2# and 3# cells compared with H1. Blue, DAPI; Green, OCT4. Scale bar: 50 μm. (C) RT‐qPCR analysis of the indicated gene expression of the H1‐*GFI1* ^*−/−*^ 1#, 2# and 3# cells compared with H1. These data represent mean ± SD from three independent replicates (*n* = 3). (D) The karyotype of H1‐*GFI1* ^*−/−*^ 1#, 2# and 3# cell lines. (E) The morphology of three germ layers by teratoma analysis from H1‐*GFI1* ^*−/−*^ 1#, 2# and 3# to show multilineage differentiation potential. Scale bar: 100 μm

**FIGURE S2** (A) FACS analysis of the classical pluripotent marker expression (OCT4, SSEA4, TRA‐1‐60 and TRA‐1‐81) of H1‐*GATA2* ^*w/eGFP*^ and H1‐*GATA2* ^*w/eGFP*^‐*GFI1* ^*−/−*^. (B) Immunofluorescence staining analysis of the classical pluripotent marker expression (OCT4) of H1‐*GATA2* ^*w/eGFP*^ and H1‐*GATA2* ^*w/eGFP*^‐*GFI1* ^*−/−*^. Blue, DAPI; Green, OCT4. Scale bar: 50 μm. (C) RT‐qPCR analysis of the indicated gene expression of the H1‐*GATA2* ^*w/eGFP*^ and H1‐*GATA2* ^*w/eGFP*^‐*GFI1* ^*−/−*^. These data represent mean ± SD from three independent replicates (*n* = 3). (D) The karyotype analysis of H1‐*GATA2* ^*w/eGFP*^ and H1‐*GATA2* ^*w/eGFP*^‐*GFI1* ^*−/−*^. (E) The morphology of three germ layers by teratoma analysis from H1‐*GATA2* ^*w/eGFP*^ and H1‐*GATA2* ^*w/eGFP*^‐*GFI1* ^*−/−*^ to show multilineage differentiation potential. Scale bar: 100 μm

**FIGURE S3** (A) WB analysis of the Akt and pAkt(Ser473) from the indicated group at day 8 of the haematopoietic differentiation (*left*). FACS analysis of the CD43+ HPC generation from the indicated group at day 8 of the haematopoietic differentiation (*right*). (B) FACS analysis of the CD43+ HPCs derived from H1‐GATA2^w/eGFP^‐GFI1^−/−^‐GFI1(FUW)‐Dox and H1‐GATA2^w/eGFP^‐GFI1^−/−^‐GFI1(FUW) + Dox cells at day 6 of the haematopoietic differentiation. Statistics was determined using unpaired two‐tailed Student's *t*‐tests; ***p < .001. These data represent mean ± SD from three independent biological replicates (*n* = 3). (C) RT‐qPCR analysis of the indicated gene expression at day 4 of the haematopoietic differentiation in H1 and H1‐GFI1^−/−^ cells. These data represent mean ± SD from three independent replicates (*n* = 3)

**TABLE S1** Primers and oligonucleotides used in this study

**TABLE S2** Antibodies and stains used for immunocytochemistry/flow‐cytometry

## Data Availability

Data sharing is not applicable to this article as no new data were created or analyzed in this study.
