# Supplementary figures and images for: GFI1 regulates chromatin state essential in human endothelial‐to‐haematopoietic transition
Source: Cell Prolif. 2022 May 3;55(5):e13244. doi: 10.1111/cpr.13244 (PMC9136496; doi:10.1111/cpr.13244)

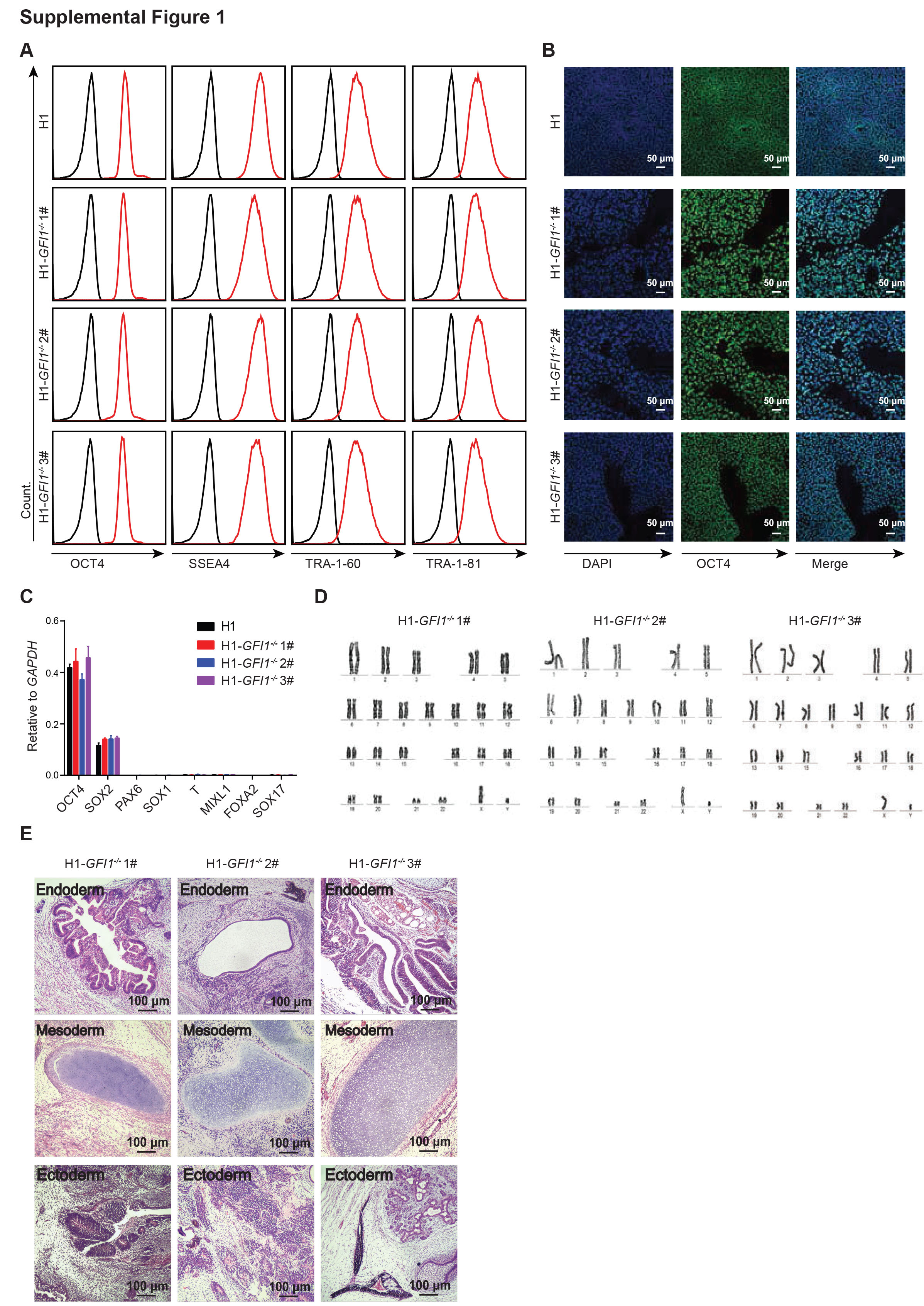

Supplement: Supplementary file 1 — FIGURE S1 (A) FACS analysis of the classical pluripotent marker expression (OCT4, SSEA4, TRA‐1‐60 and TRA‐1‐81) of the H1‐GFI1 −/− 1#, 2# and 3# cells compared with H1. (B) Immunofluorescence staining analysis of the classical pluripotent marker expression (OCT4) of the H1‐GFI1 −/− 1#, 2# and 3# cells compared with H1. Blue, DAPI; Green, OCT4. Scale bar: 50 μm. (C) RT‐qPCR analysis of the indicated gene expression of the H1‐GFI1 −/− 1#, 2# and 3# cells compared with H1. These data represent mean ± SD from three independent replicates (n = 3). (D) The karyotype of H1‐GFI1 −/− 1#, 2# and 3# cell lines. (E) The morphology of three germ layers by teratoma analysis from H1‐GFI1 −/− 1#, 2# and 3# to show multilineage differentiation potential. Scale bar: 100 μm [file CPR-55-e13244-s004.jpg]

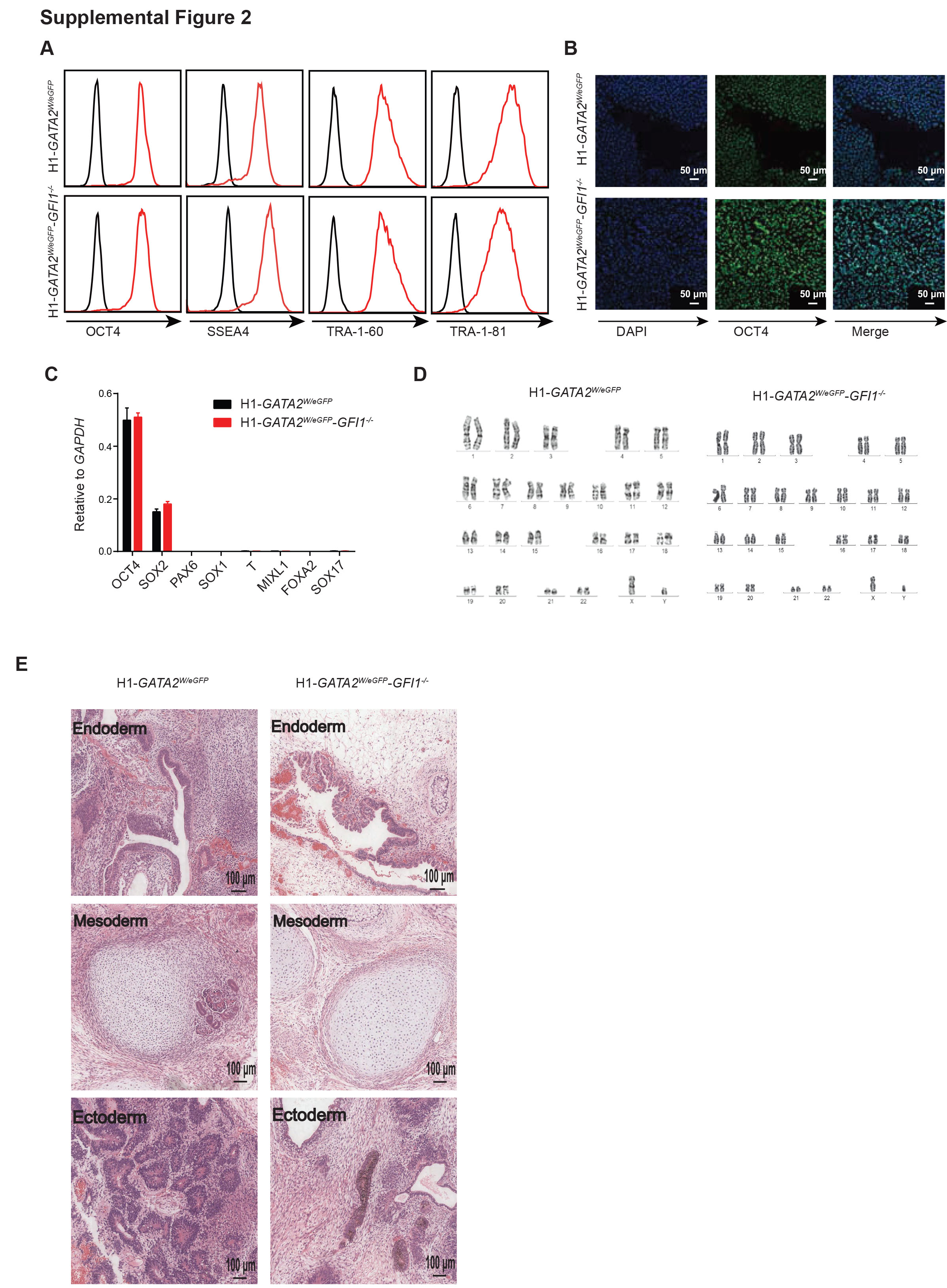

Supplement: Supplementary file 2 — FIGURE S2 (A) FACS analysis of the classical pluripotent marker expression (OCT4, SSEA4, TRA‐1‐60 and TRA‐1‐81) of H1‐GATA2 w/eGFP and H1‐GATA2 w/eGFP ‐GFI1 −/− . (B) Immunofluorescence staining analysis of the classical pluripotent marker expression (OCT4) of H1‐GATA2 w/eGFP and H1‐GATA2 w/eGFP ‐GFI1 −/− . Blue, DAPI; Green, OCT4. Scale bar: 50 μm. (C) RT‐qPCR analysis of the indicated gene expression of the H1‐GATA2 w/eGFP and H1‐GATA2 w/eGFP ‐GFI1 −/− . These data represent mean ± SD from three independent replicates (n = 3). (D) The karyotype analysis of H1‐GATA2 w/eGFP and H1‐GATA2 w/eGFP ‐GFI1 −/− . (E) The morphology of three germ layers by teratoma analysis from H1‐GATA2 w/eGFP and H1‐GATA2 w/eGFP ‐GFI1 −/− to show multilineage differentiation potential. Scale bar: 100 μm [file CPR-55-e13244-s003.jpg]

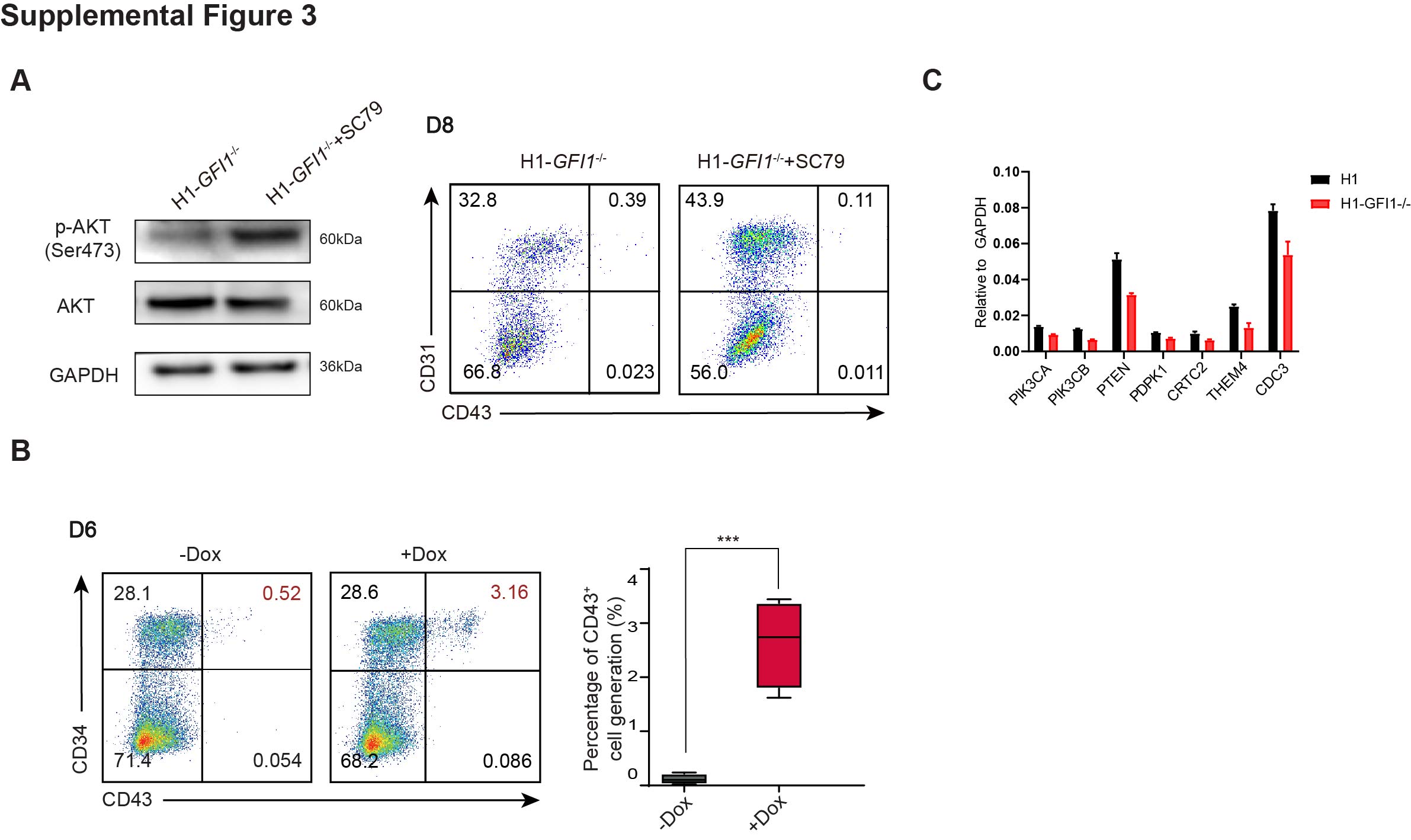

Supplement: Supplementary file 3 — FIGURE S3 (A) WB analysis of the Akt and pAkt(Ser473) from the indicated group at day 8 of the haematopoietic differentiation (left). FACS analysis of the CD43+ HPC generation from the indicated group at day 8 of the haematopoietic differentiation (right). (B) FACS analysis of the CD43+ HPCs derived from H1‐GATA2w/eGFP‐GFI1−/−‐GFI1(FUW)‐Dox and H1‐GATA2w/eGFP‐GFI1−/−‐GFI1(FUW) + Dox cells at day 6 of the haematopoietic differentiation. Statistics was determined using unpaired two‐tailed Student's t‐tests; ***p < .001. These data represent mean ± SD from three independent biological replicates (n = 3). (C) RT‐qPCR analysis of the indicated gene expression at day 4 of the haematopoietic differentiation in H1 and H1‐GFI1−/− cells. These data represent mean ± SD from three independent replicates (n = 3) [file CPR-55-e13244-s001.jpg]
