# Supplementary material for: GFI1 regulates chromatin state essential in human endothelial‐to‐haematopoietic transition
Source: Cell Prolif. 2022 May 3;55(5):e13244. doi: 10.1111/cpr.13244 (PMC9136496; doi:10.1111/cpr.13244)
Supplement: Supplementary file 4 — TABLE S1 Primers and oligonucleotides used in this study [file CPR-55-e13244-s005.docx]

**Supplemental Table 1**

| **Primers and Oligonucleotides used in this study** | |
| --- | --- |
| **Primer** | **Forward/Reverse primer (5′-3′)** |
| GFI1-sgRNA-1 | CACCCCGTTTAGAGAATGTACCGG/AAACCCGGTACATTCTCTAAACGG |
| GFI1-sgRNA-2 | CACCAGTGTCTGAGTGGATAAGCA/AAACTGCTTATCCACTCAGACACT |
| qPCR-T-F/R | TATGAGCCTCGAATCCACATAGT/CCTCGTTCTGATAAGCAGTCAC |
| qPCR-TAL1-F/R | AGCCGGATGCCTTCCCTAT/ GGGACCATCAGTAATCTCCATCT |
| qPCR-MYB-F/R | GAAAGCGTCACTTGGGGAAAA/ TGTTCGATTCGGGAGATAATTGG |
| qPCR-GFI1-F/R | GTGAGCCTGGAGCAGCACAAA/TTGCCGCACACCTGGCA |
| qPCR-OCT4-F/R | CCTCACTTCACTGCACTGTA/CAGGTTTTCTTTCCCTAGCT |
| qPCR-SOX2-F/R | CCCAGCAGACTTCACATGT/CCTCCCATTTCCCTCGTTTT |
| qPCR-SOX17-F/R | CGCACGGAATTTGAACAGTA/ GGATCAGGGACCTGTCACAC |
| qPCR-MSXL1-F/R | TCCGCAAACACAAGACGA/ ACTGCTTCTGGCGGAACTT |
| qPCR-FOXA2-F/R | ACTACCCCGGCTACGGTTC/ AGGCCCGTTTTGTTCGTGA |
| qPCR-PAX6-F/R | ATGTGTGAGTAAAATTCTGGGCA/GCTTACAACTTCTGGAGTCGCTA |
| qPCR-SOX1-F/R | AATTTTATTTTCGGCGTTGC/TGGGCTCTGTCTCTTAAATTTGT |
| qPCR-GAPDH-F/R | GTGGACCTGACCTGCCGTCT/GGAGGAGTGGGTGTCGCTGT |
| qPCR-LMO2-F/R | GGCCATCGAAAGGAAGAGCC/ GGCCCAGTTTGTAGTAGAGGC |
| qPCR-GATA2-F/R | ACTGACGGAGAGCATGAAGAT/ CCGGCACATAGGAGGGGTA |
| qPCR-RUNX1-F/R | TGAGCTGAGAAATGCTACCGC/ ACTTCGACCGACAAACCTGAG |
