# Supplementary material for: GFI1 regulates chromatin state essential in human endothelial‐to‐haematopoietic transition
Source: Cell Prolif. 2022 May 3;55(5):e13244. doi: 10.1111/cpr.13244 (PMC9136496; doi:10.1111/cpr.13244)
Supplement: Supplementary file 5 — TABLE S2 Antibodies and stains used for immunocytochemistry/flow‐cytometry [file CPR-55-e13244-s002.docx]

**Supplemental Table 2**

| **Antibodies and stains used for immunocytochemistry/flow-cytometry** | |
| --- | --- |
| **Antibody** | **Company and Cat #** |
| PE Mouse anti-human CD31 | BD biosciences Cat#555446 |
| PerCP/Cyanine5.5 anti-human CD34 | Biolegend Cat#343522 |
| APC Mouse anti-human CD43 | BD biosciences Cat#560198 |
| Mouse Anti-Hemoglobin β (37-8) | Santa Cruz Biotechnology Cat#: sc-21757 |
| Mouse anti-OCT3/4 | Santa Cruz Biotechnology Cat#:sc-5279 |
| Mouse anti-SSEA4 | Santa Cruz Biotechnology Cat#: sc-21704 |
| Alexa Fluor® 647 Mouse anti-TRA-1-60 | BD biosciences Cat# 560850 |
| Alexa Fluor® 647 Mouse anti-TRA-1-81 | BD biosciences Cat# 560793 |
| Alexa Fluor 488 Goat anti-Mouse IgG | Thermo Fisher Scientific Cat# A-11001 |
| Alexa Fluor 647 Goat anti-Mouse IgG | Invitrogen Cat# A-21235 |
